# 

**Published:** 1876-08

**Authors:** 


					﻿[Vol. XVI]
AJJGUST, 1876.
[No. l.J
BUFFALO
Webital anti Surgical found.
EDITED BY EDWARD N. BRUSH, M. D.
BUFF A.X. O^
PUBLISHED FOR THE EDITOR?
1876.
				

